# Supplementary material for: Full-Length RNA Sequencing Provides Insights into Goldfish Evolution under Artificial Selection
Source: Int J Mol Sci. 2023 Feb 1;24(3):2735. doi: 10.3390/ijms24032735 (PMC9916754; doi:10.3390/ijms24032735)
Supplement: Supplementary file 1 [file ijms-24-02735-s001.zip › ijms-2111439-supplementary.pdf]

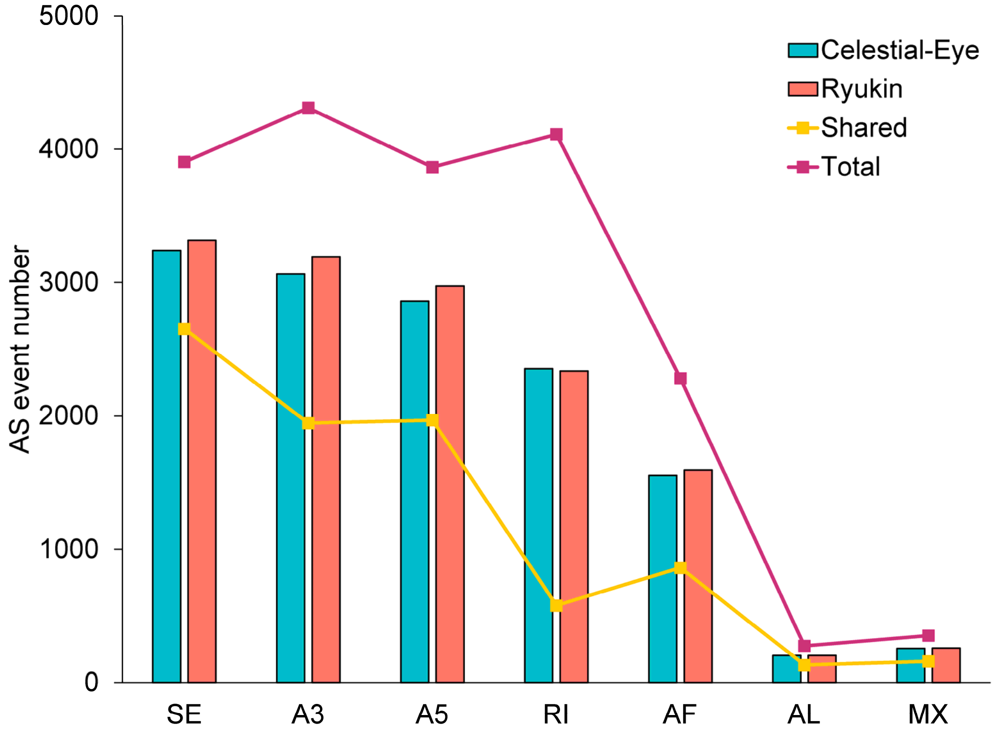

Figure S1. alternative splicing identified in Celestial-Eye and Ryukin goldfish embryos.

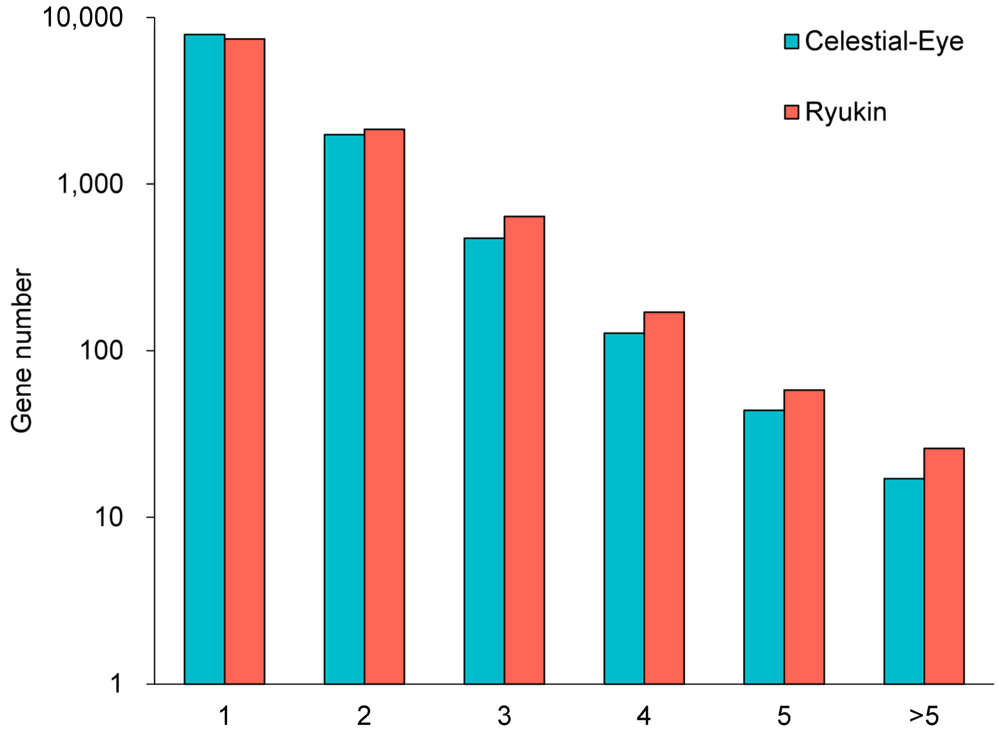

Figure S2. alternative polyadenylation identified in Celestial-Eye and Ryukin goldfish embryos.

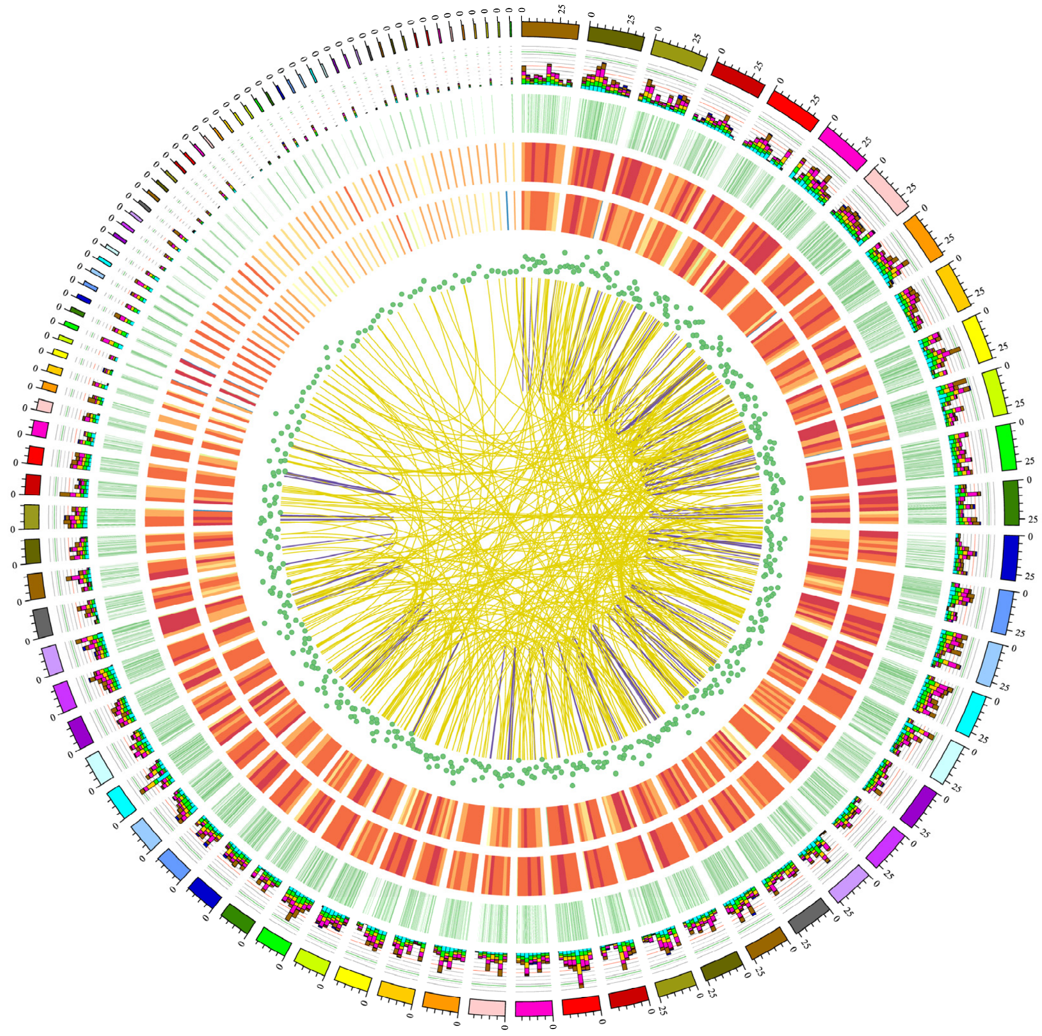

Figure S3. CIRCOS visualization of different data of Celestial-Eye at the genome-wide level.

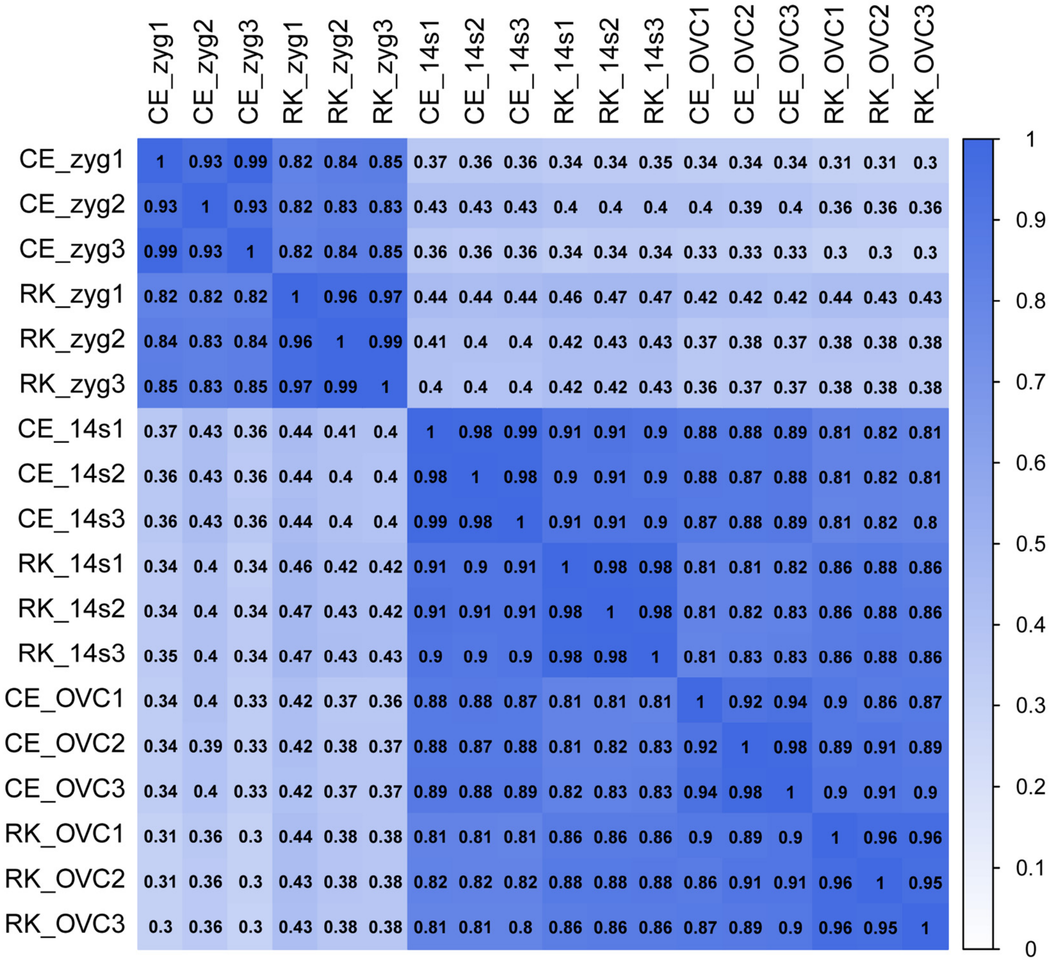

Figure S4. Pearson correlation analysis of RNA-seq data.

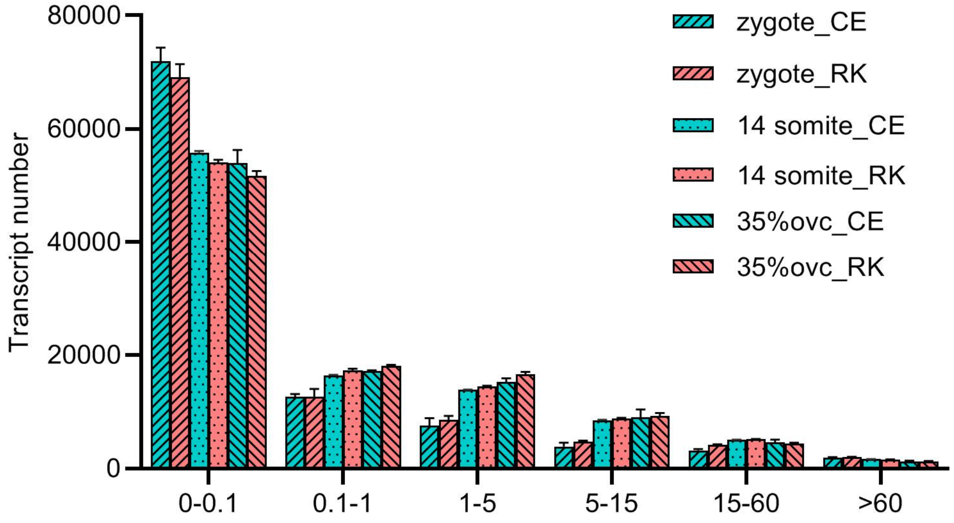

Figure S5. gene FPKM distribution of embryos at different stages.

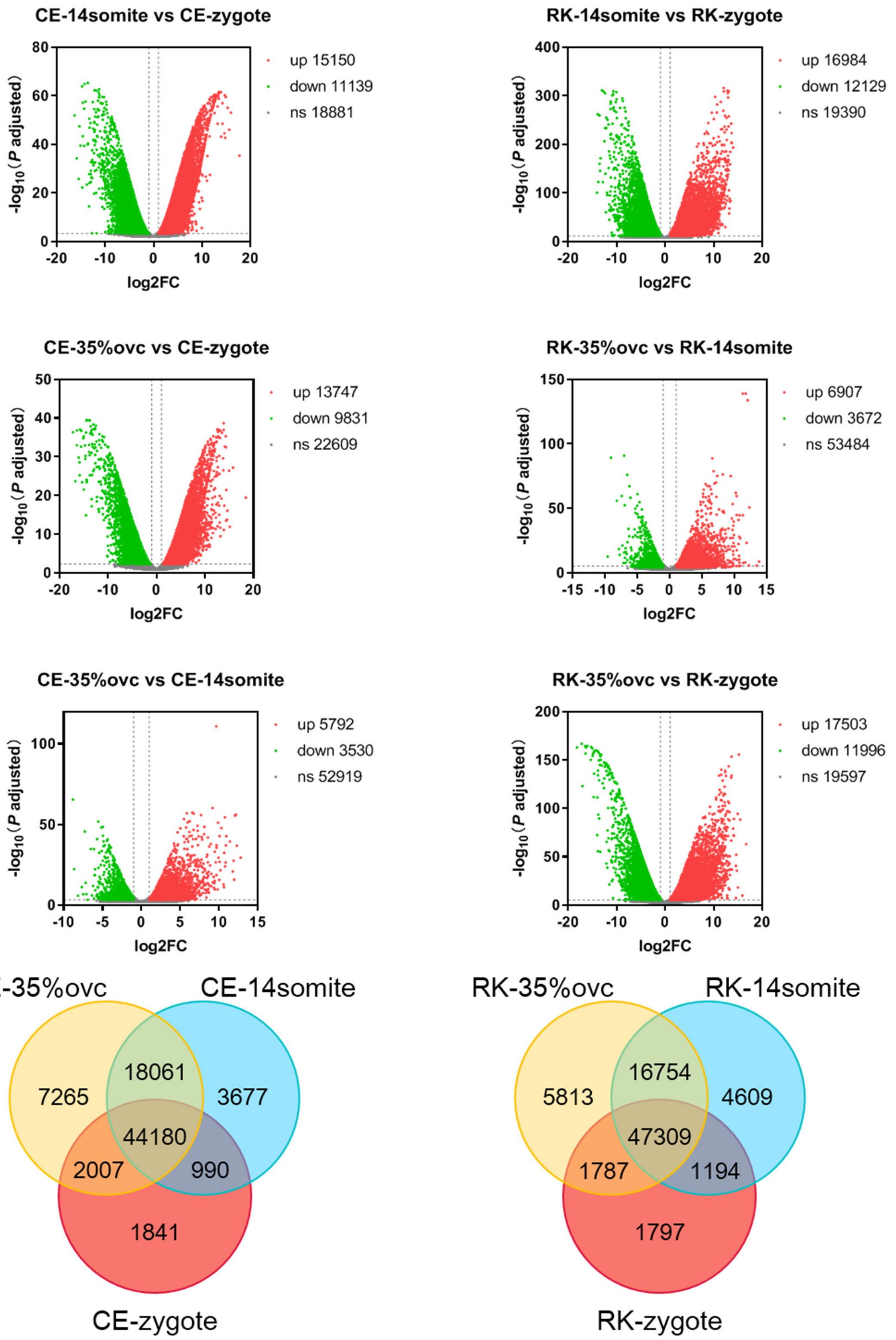

Figure S6. embryo stage specific expression of genes in Ryukin and Celes-tial-Eye goldfish.

Table S5. summary of RNA-seq data.

Sample	Total reads	Mapping rate
Celestial-Eye_zyg1	59801696	93.81%
Celestial-Eye_zyg2	46567760	92.28%
Celestial-Eye_zyg3	50524518	94.04%
Celestial-Eye_14s1	50763956	93.26%
Celestial-Eye_14s2	55641526	93.11%
Celestial-Eye_14s3	52026462	92.47%
Celestial-Eye_35%OVC1	48814838	92.99%
Celestial-Eye_35%OVC2	66470196	93.01%
Celestial-Eye_35%OVC3	50026396	93.12%
Ryukin_zyg1	71228022	93.59%
Ryukin_zyg2	52927042	93.44%
Ryukin_zyg3	61859812	93.66%
Ryukin_14s1	63156658	92.91%
Ryukin_14s2	57689608	93.21%
Ryukin_14s3	44621642	92.54%
Ryukin_35%OVC1	50001060	92.27%
Ryukin_35%OVC2	45820876	91.91%
Ryukin_35%OVC3	59051054	94.65%
